# Cherish your children: socio-economic and demographic characteristics associated with child mortality

**DOI:** 10.1186/s12889-021-11276-9

**Published:** 2021-06-24

**Authors:** Ruwan Jayathilaka, Harindu Adikari, Rangi Liyanage, Rumesh Udalagama, Nuwan Wanigarathna

**Affiliations:** 1 Department of Information Management, SLIIT Business School, Sri Lanka Institute of Information Technology, New Kandy Road, Malabe, Sri Lanka; 2 Department of Business Management, SLIIT Business School, Sri Lanka Institute of Information Technology, New Kandy Road, Malabe, Sri Lanka

**Keywords:** Child mortality, Socio-economic and demographic characteristics, Logit

## Abstract

**Background:**

The United Nations Interagency Group for Child Mortality Estimation (UNIGME) indicates that child mortality is the death rate of children between age zero to five. The importance of this area of research is high where worldwide a number of studies have been led on infant and child mortality, despite limited research discoveries with regards to Sri Lanka. The aim of this study is to investigate the socio-economic and demographic characteristics associated with child mortality in Sri Lanka.

**Methods:**

Using the context of Sri Lanka as a case study, this study carried out based on data gathered from the micro level national survey. Using the logit regression model through the step-wise technique, the study investigate the socio-economic and demographic characteristics associated with child mortality in Sri Lanka.

**Results:**

According to the generated results, place of residence province-wise, household head’s education level and source of drinking water have negative effect (lower risk) on child mortality in Sri Lanka. Exceptionally, the Western province has the highest negative effect on child mortality which demonstrates it as the least harmful region in Sri Lanka in child endurance. Household heads who owns private entities and Sri Lankan Moors has a positive effect on child mortality as well.

**Conclusion:**

This study is helpful to address the population health of local arena and results can be supportive to the government and policymakers to gain an overview of physical health status of the country and able to uplift their policies based on the new findings.

## Background

Children can be considered as significant to any nation, who will be the future generation. It is therefore necessary to have a critical investigation into child mortality, as to whether a country’s posterity is adequately secured during their early years of growth. To review child mortality and its effective establishment, past analysts have typically used monetary and section attributes and individual level characteristics [1]. According to the United Nations Interagency Group for Child Mortality Estimation (UNIGME), deaths of children during the age 0–5 for each thousand live births show the level of child mortality. This child mortality can be known as infant mortality, child death rate and under-five mortality [2].

The World Health Organization (WHO) discloses that in 1990, 12.6 million child deaths occurred each year over the world which has decreased to 5.6 million in 2016 [3]. These statistics exhibit a notable progress in child prosperity, where decline in death rates have limited child mortality. Similarly, it shows that 5.3 million of child deaths were reported in 2019 and further explains that 15,000 child deaths take place each day over the world. Among these, 2.5 million are deaths below the age of 1 month. With globalisation and easy accessibility to data, it cannot be accepted that majority of these deaths are due to treatable sicknesses or avoidable causes [4]. Besides, half of the world child deaths were reported consistently in six countries namely: China, India, Pakistan, Nigeria, Ethiopia, and Demographic Republic of Congo. In generally, predominant aspect of the child deaths happened in Sub-Saharan Africa and South Asian countries [5].

Child mortality rate of Sri Lanka fell drastically from 70.1 deaths per 1000 live births in 1970 to 7.1 deaths per 1000 live births in 2019. However, the future human capital for any country is children. As researchers, we have identified the importance of conducting a study which aims to capture socioeconomic and demographic behaviour on child mortality in Sri Lanka. Thereby, this research aims to provide findings that can address issues to reduce child mortality.

Moreover, the Department of Census and Statistics of Sri Lanka (DCS) highlighted that the infant mortality or early childhood mortality is a measure of socioeconomic development of a country, which is also a valuable yardstick to measure quality of life. Furthermore, the highest number of under-five deaths take place in the *Kilinochchi* district and the lowest in the *Polonnaruwa* district. However, the estate sector has somewhat a higher possibility of children dying before reaching the age of one than children in the urban and rural sectors. As for differentials expected in infant and child mortality, education level of the mother and household wealth play a major role. In terms of demographic characteristics which affect infants and child deaths, the gender of the child, mother’s age at birth, birth interval and the birth order are identified as the most important characteristics.

Many rigorous studies have been conducted on child mortality and household income and expenditure, household wealth and socioeconomic determinants of child mortality, especially in the developing countries in Asian and African regions. However, in Sri Lanka, in the cent past, it is hard to find empirical evidence pertaining to infant and child mortality. As a low-middle income country with a developing economy, this area of research is of significance to Sri Lanka. This study is an opportunity to address public health issues and device solutions in this regard. In addition, the study provides insights both to identify socioeconomic and demographic characteristics as well as association of a household which had experienced child deaths.

Numerous detailed examinations on child mortality including socio-economic and demographic characteristics, attributes of families and financial determinants of child mortality have been conducted particularly in developing nations like Asian and African regions. Nevertheless, limited studies in this area of research based on Sri Lanka means that research gaps can also be observed. By understanding the socio-economic and demographic characteristics associated with health concerns, Sri Lanka as a developing low-middle income country combatting the threat of coronavirus (COVID-19) pandemic, can benefit from these study findings to proactively and effectively deal with health issues.

Consequently, child mortality is a pointer of general wellbeing. As a health indicator, it assigns a higher importance criticalness for developing economies like Sri Lanka regarding this area of examination (to distinguish their degree of wellbeing for developing economies on how financial and segment attributes influence child death mortality). Besides, constraints can be observed in past research studies conducted by numerous specialists in various nations.

Geologically, most under five deaths occur only in two areas of the world [6]. In 2017, among under five deaths, half (50%) occurred in sub-Saharan Africa while 30% in Southern Asia. Accordingly, researches which focus subject areas like infant and child mortality is rather limited in a Sri Lankan setting (as noted in Section 1). As a result, research findings addressing socio-economic and demographic characteristics of child mortality are rare in the Sri Lankan context [7].. Thus, this investigation assists to identify the socio-economic and demographic characteristics associated with households in Sri Lanka towards child mortality. In doing so, the present study helps bridge the research gap in prevailing in a local setting.

Research question can be expressed as “what is the nature of the of socio-economic and demographic characteristics on child deaths?”. This exploration is a massive incentive to strategy creators and policymakers of Sri Lanka, as a centre of a low pay nation and developing economy. Time is appropriate to address how the economic background of household is related to child mortality in a developing country like Sri Lanka. As a South-Asian country which has had over a 20 year civil war and due to lack of research findings in the local arena, this study is significant in the context of Sri Lanka. By addressing socioeconomic and demographic characteristics pertaining to child death through findings of this research, responsible authorities and stakeholders have access to relevant information much easily. Accordingly, authorities and policymakers can duly modify the respective framework, with the aim of minimising child deaths.

Present study’s literature search is based on initially identified 212 publications during 1973–2019, in reputable journal databases such as Science Direct, Emerald Insight, Elsevier, Springer, JSTOR, and Research Gate etc. In searching articles, keywords such as child mortality, householders and child survival were used.

**Fig. 1:**
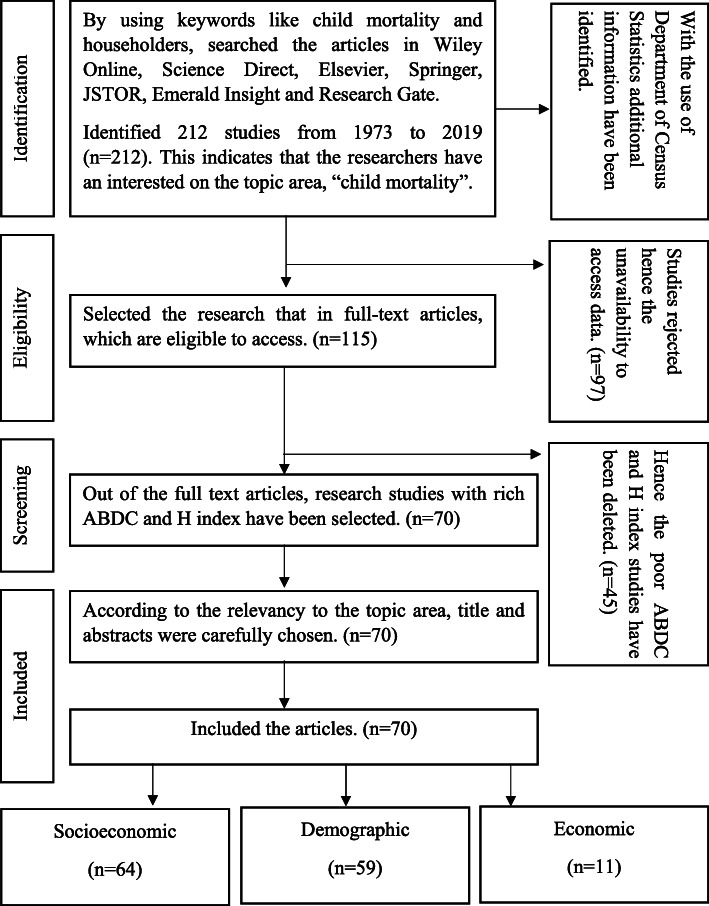
Literature search flow diagram. Source: Based on authors’ observations

Thereafter, a screening procedure was carried out including the most relevant publications which fulfil requirements of the literature review. Consequently, 115 papers qualified for full-text access. The remainder 97 articles were refused, due to unavailability to access details. Figure 1 the flow diagram displays the number of studies found, preserved, and discarded at each point of the literature review.

Accordingly, out of the full text articles, 70 studies have been shortlisted due to the reason of having rich Australian Business Deans Council (ABDC) ranking and H index. At this point, with the help of keywords that have already been used, along with advanced content and methods incorporating contemporary literature on the subject area, these studies were regarded as highly applicable to the selected area of research.

Finally, the literature review of this study was established based on 70 research articles papers. This provides a detailed picture of the study using previous studies carried out in different countries around the world on infant mortality. Furthermore, the above mentioned 70 papers are subcategories into three, based on variables discussed pertaining to the study as: socio-economic variables, demographic variables, and economic variables. Most notably, several studies have been duplicated, therefore, studies have been targetted at various factors or finding various infant mortality relationships. Figure 1 shows the flow of this development of the literature.

Over the last two centuries, seemingly countries around the world have made rapid progress against child mortality. Global mortality has halved from about 43% to 22.5% from 1800 to 1950. The mortality rate has declined five-fold from 22.3 in 1950 to 3.8% in 2019. This development has spread its benefits worldwide. Globally, 3.9% of children die before they reach the age of five, meaning that an average of 15,000 children die every day. From year 1990 to 2016, child death rate has dropped by 7 million. Researchers indicates that the growth of each country’s socio-economic history is the reason behind the deduction of child mortality [3, 8].

Sub-Saharan Africa is the region with the highest child mortality rate in 2017; 76 child deaths per 1000 live births or it can be described as 1 child death in 13 live birth before their fifth birthday due to environmental factors such as family background or living in a deprived community background. According to the United Nations (UN) in 2018, 50% child deaths were reported from Sub-Saharan Africa whereas 30% from South Asia. In addition, UN also declared that the poorest households are more at risk than rich households with regard to child mortality. This evidence highlighted that child mortality is of immense importance to Sri Lanka due to being a growing economy in Southern Asia.

In terms of Sri Lankan context, the rate of infant mortality varies between socio-economic groups. However, important factors related to infant mortality are associated with health issues, hunger issues and the level of education of women [9]. Furthermore, J Trussell and C Hammerslough [7] found that the degree of parental instruction, home place, ethnicity, gender of young people, age of the mother at the birth, and accessibility to hygienic latrine facilities have a noteworthy impact on child mortality. S Rajindrajith, S Mettananda, D Adihetti, R Goonawardana and NN Devanarayana [10] are of the view that Sri Lanka’s major cities as well as the estate sector face substantial risk of infant deaths; also households in these regions are likely to experience complications of preterm birth, neonatal sepsis and cardiac defects that cause neonatal deaths. The present study intends to discuss the current situation of infant deaths with associated socio-economic and demographic characteristics, according to the Household Income and Expenditure Survey (HIES) 2016. Further, a detailed analysis of this kind provides additional value for users of information and stakeholders targetting effective decision making that can minimise child mortality. Meanwhile, savings the lives of children aged 1–4 is highly required in low-income countries where the mortality rate are usually high.

Many significant socio-economic factors influencing child mortality can be observed such as place of residence, households head’s education level, sources of drinking water, available toilet facilities and household head’s employability status.

Place of residence plays an important role in determining infant mortality when considering urban and rural areas [4]. This findings is much valid among developing countries, where remote areas have limited resources. Urban areas, congested living, high vehicle emission rates and availability of industrial facilities aggravate child mortality [4]. Child mortality among people in urban areas in developing countries is lower than that among rural areas, largely because of access to facilities. Findings confirmed that the community level infrastructure available for healthcare services will be limited in rural areas opposed to urban areas. Furthermore, regression results also report that significant differences exist between regions in Turkey [11]; this finding indicates that mothers living in the south-western part of Turkey have experienced lower infant mortality rates even after adjusting for characteristics of socio-economic, population level (such as area and urban-rural residence) and household level. It has been noted that the inclusion of the index of household wealth makes the difference of urban-rural residence negligible. However, re-estimating the model by excluding the asset index, the coefficient for urban residence is statistically significant; mothers residing in urban areas experienced fewer infant deaths than mothers residing in rural areas. However, when the asset indices are included as regressors, the urban variable is no longer significant.

Households head’s education level is another socio-economic variable that associate on child mortality. Past evidence according to literature addressing education level of the mother, education level of the father and the level of education of the household head have been discussed in this study. Kaberuka et al. (2017) emphasised that there is higher risk for child mortality when households head is educated up to primary level compared to household heads who had received educated secondary and above [7]. asserted that a father’s level of education also significantly associate with child mortality in Sri Lanka. Additionally, this research has proved that highly educated men have a low risk of infant death or level of education of the father has a negative effect on child mortality. Extensive research conducted by [12] stress that although mothers who never had any sort of education have a high risk of having a child death during their lifetime, parents with higher education level is more likely to have a low child mortality rate. In other words, educated parents are more knowledgeable and capable of handling child health issues. Moreover, education of parents (both mother’s and father’s education) significantly associate with health issues of children [13]. The greater the education received by parents, the lower the infant mortality rate. According to the regression of this study, a mother with secondary education has fewer infant deaths than a mother who has never received education.

It is evident from estimates reported in this study that household economic well-being has substantial effects on infant mortality. Even after controlling the impact of the variable for mother’s and father’s education and also the richest mothers have fewer infant deaths than that of the poorest mothers [11]. Findings of U Iram and S Butt Muhammad [14] show that the level of education of mothers has a negative and powerful effect on neonatal and infant mortality without any noticeable impact on the mortality of infants moreover; the outcome means that relatively properly qualified mothers are better able to understand the health needs of their children and can provide better care for their children. W Kaberuka, A Mugarura, J Tindyebwa and D Bishop [15] emphasised that Children born to mothers of secondary and higher level of education have reduced risks of dying before age five when compared to those who are born to mothers with no education (OR = 0.349, *p* = 0.014). This is consistent with research stating that education equips women with knowledge about health and additionally influences their attitude toward proper nutrition and health.

Nevertheless, source of drinking water are another socio-economic variable that influences child mortality. In many developing countries, lack of access to clean water is more vulnerable to health disorders. In 2002, the United Nations Children’s Fund (UNICEF) study reported that more than half of the world’s population used pipeline water at home. In addition, enhanced drinking water supplies are used by 92% of the urban population and 70% of the rural population in developing countries. There may be many factors behind the highest infant death rate during the neonatal era, such as sanitation, public health services, and access to safe drinking water; the study claims the neonatal mortality rate among newborns has been found to be higher for mothers in households with access to no clean drinking water [16]. Reviews conducted in Zimbabwe [17] emphasised that availability of piped drinking water in the dwelling shows a negative impact on child mortality; though alpha is not significant for children born in households with access to piped drinking water, the risk of dying during childhood has decreased by 39% compared to those born in households without access to piped drinking water.

Findings in many cases revealed that availability of toilet facilities in home as an indicator of the level of sanitisation; toilet sanitation have been found to be associated with child mortality in a variety of locales. Type of toilet facilities in the household has a significant association on child mortality. Access to a flush or pit toilet for infant mortality has a clear influence on child survival [18]. Families which use modern flush toilets are found to be associated with the lowest infant mortality, where pan toilets tend to be safer than pit latrines and private toilets for child survival, than public ones. The relative risk of death for children is high relative to children born in households with no access to improved toilets and less toilets with less facilities compared to households with improved toilet facilities [17]. Availability of improved toilet facilities on child mortality also in the negative direction, significantly affect to reduce child death rates [17]. The relative risk of death for children born in households with access to improved toilets is decreased by 60% in comparison to children born in households without access to improved toilets (*p* < 0.01).

Moreover, household head’s employability status is another socio-economic variable that can highly affect child mortality. A research specifies that the improvement in employment levels and economic growth will lead to reduce child mortality [19]. This fact is further confirmed by empirical evidences of another research. Here, parental occupation is related to nutritional status of their children and the occurrence of infant and child mortality is higher among nonworking parents including those engaged in regular household activities, than parents who are working; thus, it determines the economic status, housing condition, access to health care facilities, nutrition and clothing of a family as factors affecting child mortality. Furthermore, results indicate that neonatal mortality is high among farmers and businessman [20].

Some studies claim that literature of past studies reflects the effect of different demographic characteristics on child mortality such as household head’s age, religion, and ethnicity. Furthermore, demographic data on child mortality is useful to identify the future population health of a country while identifying areas to be developed or more resources to be allocated, based on past behaviour of demographic characteristics in a given country.

Most research demonstrate that the age of the mother at birth of a child is an important factor which can be linked with the risk of dying. Women who have given birth at an older age are susceptible to complications; U Iram and S Butt Muhammad [14] defines that neonatal women are also at an increased risk of premature birth. Late marriage continues to increase the risk of child death due to weak reproductive system and less stability to control difficulties of childbirth. In Sri Lankan setting, the young mothers and older mothers face higher dangers of dying on child birth than those in the 20–34 age group. J Trussell and C Hammerslough [7], show that death rates for children born to mothers who are less than 20 years of age are considerably greater than children born to mothers aged 20–34. Furthermore, children born to older mothers aged above 35 also adversely impact on child mortality. A thought provoking fact in a study conducted in Uganda W Kaberuka, A Mugarura, J Tindyebwa and D Bishop [15] emphasised that children born to mothers in the age group from 40 to 49 have increased risks of dying before reaching the age of five when compared to those born to mothers in age group 15–19 (OR = 1.621, *p* = 0.006). It was observed that the likelihood increased with the increasing age of the mother. Children born to a mother between 30 and 39 years of age have the least risk of dying before the age five (OR = 0.865, *p* = 0.014).

Religion of the household is another demographic factor associated with child mortality, which can be interpreted as the belief and adoration of a superhuman power of control, especially a personal power of control. Evidence has consistently shown that religion is a risk factor which affects child mortality [21]. The study conducted by C Pörtner, F Tarp and J Kovsted [22] confirms that mothers’ religion was an important factor when determining the health of the child. Furthermore, in the study conducted by A Pandey [23] in India, the relationship between child mortality and religion of households with regard to child deaths was described as positive. Another study claims that most religious institution provides free education through seminars which helps improve knowledge related to child mortality in way that empower their living standards; mothers religion are found to have is associate with lower child mortality rates [24]. Sri Lanka being multi-cultural, the demographic feature of ‘religion’ is important to note.

Ethnicity is a diverse term associated with differences in health attitudes and behaviours that includes culture, lifestyle, language, and ethnicity. Ethnicity is another variable associated with child mortality. Diverse are cultures and ethnicity in countries and each of these variables affects how rates of child mortality vary from each type [25]. The finding also emphasises on perinatal and infant mortality in a study performed in the Netherlands; accordingly, the study concluded that African mothers had the highest rate of perinatal mortality compared to white mothers in the Netherlands [26]. Additionally, another study affirms that, the nation has a diverse geographical and socio-political context and has multiple cultural and linguistic communities in it [27]. Nigeria is Africa’s most populated region, by large. Despite significant improvements in health outcomes for children in the last century, under-five mortality in Nigeria remains unacceptably high.

The economic characteristics of the household may differ according to the structure, model, and behaviour of the level of household income and expenditure. Confirming the above fact, a study claims that the parental income level is of positive significance with relating to child mortality in many Western countries [15]; parental income has the ability to spend money on healthcare services and medicine which is important especially for the children who is in the 1–5 age category [5]. More the healthcare and medication expenditure, the more likely to survive from infections and different forms of infections which are essential for children’s immune system, hygiene and nutrition [28]. Further, W Kaberuka, A Mugarura, J Tindyebwa and D Bishop [15] show that children born to a mother from a wealthy family are 0.8 times less likely to die before 5 years of age (OR = 0.441, *p* = 002) than children born to mothers from poor families. Children born to a mother from an average income family have reduced risks of dying before 5 years of age compared to children born to a mother from a poor family (OR = 0.650, *p* = 0.024).

In Sri Lanka, the child mortality rate differs among socio-economic groups. Nevertheless, problems related to healthcare, malnutrition and level of education among females have been identified as significant factors related to child mortality [9]. Similarly J Trussell and C Hammerslough [7] have found that parental education level, place of residence, ethnicity, child’s sex, age of mother at birth and availability of toilet facilities have been significantly associated with child mortality. S Rajindrajith, S Mettananda, D Adihetti, R Goonawardana and NN Devanarayana [10] show that major cities in Sri Lanka and the estate sector face significant risk on child deaths and to complications associated with preterm birth, neonatal sepsis and cardiac anomalies. Latter are identified as significant causes for neonatal deaths as well.

Almost all the literature review above indicated a separate aspects of socio-economic and demographic characteristics on child mortality. Table 1 represents some of the variables related to past research studies of socio-economic and demographic characteristics on child mortality. Thus, this study will focus to contribute to this empirical gap by examining how the socio-economic and demographic characteristics are associated with child mortality as a whole.

**Table 1:** Summary of literature: Variables and supporting research articles

Variable	Research Papers
*Child Mortality*	AM Veneman [2], U Iram and S Butt Muhammad [14], JR Khan and N Awan [21], T Houweling, C Ronsmans, O Campbell and A Kunst [9], J Trussell and C Hammerslough [7], S Rajindrajith, S Mettananda, D Adihetti, R Goonawardana and NN Devanarayana [10].
*Socio-economic Characteristics*	
Place of Residence	O Ezeh, K Agho, M Dibley, J Hall and A Page [4], SK Gaisie [29], A Genowska, J Jamiolkowski, K Szafraniec, U Stepaniak, A Szpak and A Pajak [30], IN Abu, IA Madu and CK Ajaero [31] H Yanıkkaya and S SelİM [13].
Income	L Mogford [5], W Kaberuka, A Mugarura, J Tindyebwa and D Bishop [15], MS Durkin, LL Davidson, L Kuhn, P O’Connor and B Barlow [32], S Ssewanyana and SD Younger [33], M Kimani [34], J Caldwell and P McDonald [28], C Nyamuranga and J Shin [35].
Household Head’s Education Level	AM Veneman [2], M Kimani [34], W Kaberuka, A Mugarura, J Tindyebwa and D Bishop [15],J Trussell and C Hammerslough [7], RK Stella Lartey, Shingo Takahashi [36], MNI Mondal, MK Hossain and K Ali [20], M Babayara and B Addo [12], PJ Kembo and J Ginneken [17], U Iram and S Butt Muhammad [14], S Rajindrajith, S Mettananda, D Adihetti, R Goonawardana and NN Devanarayana [10].
Sources of Drinking Water	Y Shiferaw, M Zinabu and T Abera [37], O Ezeh, K Agho, M Dibley, J Hall and A Page [4], UR Saha and A van Soest [38], S Rabbani and A Qayyum [16].
Available Toilet Facilities	PW Stephens [39], PJ Kembo and J Ginneken [17], W Kaberuka, A Mugarura, J Tindyebwa and D Bishop [15].
Household Head’s Employability Status	CO Odimegwu, EO Olamijuwon, VH Chisumpa, JO Akinyemi, MG Singini and OD Somefun [40], J Akinyemi, B Solanke and C Odimegwu [41], V Tripathi and R Singh [42], MNI Mondal, MK Hossain and K Ali [20].
*Demographic Characteristics*	
Household Head’s Age	U Iram and S Butt Muhammad [14], MNI Mondal, MK Hossain and K Ali [20], PJ Kembo and J Ginneken [17], RA Bello and AI Joseph [43], O Ezeh, K Agho, M Dibley, J Hall and A Page [4], Y Shiferaw, M Zinabu and T Abera [37], IN Abu, IA Madu and CK Ajaero [31], YB Okwaraji, S Cousens, Y Berhane, K Mulholland and K Edmond [44], J Trussell and C Hammerslough [7].
Religion	FV Poppel, J Schellekens and AC Liefbroer [45], JR Khan and N Awan [21], A Pandey [23], C Pörtner, F Tarp and J Kovsted [22], C Mutunga [46], R Apunda [24].
Ethnicity	CG Victora, AJD Barros, C Blumenberg, JC Costa, LP Vidaletti, FC Wehrmeister, B Masquelier, L Hug and D You [25], TWJ Schulpen, JE van Steenbergen and HF van Driel [26], M Brockerhoff and PC Hewet [47], SA Adedini, C Odimegwu, ENS Imasiku and DN Ononokpono [27].

Source: Authors’ compilation

The present addresses the current situation of child deaths with related to the socio-economic and demographic characteristics by using the HIES 2016. Scope of work entails an extensive analysis on income and expenditure of households related to child mortality. Thus, the present study findings can be an added advantage to readers and stakeholders.

The purpose of this study is to determine the socio-economic and demographic characteristics associated with child mortality. There are three main ways that this study differs from the existing studies. First, this study mainly considers the economic background of the household on child mortality. Due to this reason, the present study can be a reliable source of information useful for health sector and policymakers alike in Sri Lanka. Here, the purpose is to ensure quality of living and child survival level by utilising available resources productively, specially during a pandemic situation like COVID-19.

Second, research findings which has focussed on socio-economic and demographic characteristics on child mortality are rare and limited in Sri Lankan context [7]. This study contributes with timely findings to help bridge the research gap in this regard in investigating the current level of socio-economic and demographic characteristics associated with child mortality and how it affect child deaths during age 0–5.

Finally, this research study assists government to understand country’s unmet health needs of the country, pay due attention to less privileged segment of society in the developing economy in terms of child mortality. Likewise, it improves the readiness of the government in ensuring the population health of the country.

## Methods

Henceforth, this examination basically focusses on the socio-economic and demographic characteristics of households associated with child mortality gathered by the HIES 2016 which has been led by the DCS, Sri Lanka. This examination considers the whole populace of Sri Lanka 21.44 million (2017) gathering information in 12 months consistently from January to December in 2016; the HIES has been conducted in every region, thus, representing all (25) regions in Sri Lanka with more than 21,756 householders. In the interim, this investigation involves having households with the cross-sectional classification and does not take into account various time slots identified with child mortality.

The sample design of the investigation depends on two stage stratified sampling. Sample population has been classified into various classes as indicated by socio-economics and economics attributes, for example, mother’s age, spot of living arrangement and salary and consumption etc. According to this investigation, the principle area utilised for separation is the local and urban, rural and estate areas in each region are the choice spaces for the investigation. With regards to inspecting outline is the rundown of lodging units arranged for the statistics of populace and housing. Furthermore, selecting sample size of the district is based on proportion to the number of housing units and the standard deviation of the mean household expenditure value stated in corresponding districts in earlier surveys. Moreover, Neyman Allocation method has been used as sample allocation.

According to past evidences, conventional models such as multiple regression used to identify the socio-economic and demographic characteristics of households associated with child mortality and in recent years, mainly logit or probit regression models have been used [14]. Thus, it is sensible to accept that the logit regression is effective in this study to generate reliable results. It helps measure the dependent variable of the study which has two possibilities as; households who has experienced on child deaths and those who have not. The parameter of the model is based on the change of independent variables of the study [35].

The strategic equations are expressed as far as the likelihood that Y = 1, which is alluded to as P. The likelihood that Y is 0 is 1 - P.

1 $$ \ln \left(\frac{P}{1-P}\right)=a+ bX $$

In the eq. 1, *ln* symbol refers to a natural logarithm and *a + bX* is our familiar equation for the regression line. *P* can be figured from the relapse condition moreover. Thus, the relapse condition is known, hypothetically, normal likelihood is that Y = 1 for a given estimation of X.

2 $$ P=\frac{\exp \left(a+ bX\right)}{1+\exp \left(a+ bx\right)}=\frac{e^{a+ bx}}{1+{e}^{a+ bx}} $$

In the eq. 2, exp. is the exponent function, occasionally composed as e. Thus, the condition on the privilege is only something very similar yet supplanting exp. with e. However, there is not the lingering. You can generally tell when e represents exp. on the off chance that you see that there is a superscripted esteem with the e, proposing that e is raised to some power.

The logit model of this study has developed with the help of dummy variables as; 1 for households who has experienced on child deaths and 0 for households who has not experienced on child deaths. Table 2 indicates the explanatory variables which could affect child mortality in local arena. Those explanatory variables can be categorised as, socio-economic variables and demographic variables. The forward-wise logit regression help identify the significant variables on child mortality and this selection will be with *p*-value< 0.10 and recently chosen factors for evacuation with *p*-value > = 0.15.

**Table 2:** Variable definitions for household dataset

Variable	Description	Expected Sign (s)
**Dependent Variable**		
Child Mortality	1 if a household experienced in child deaths; 0 if not	
**Independent Variables**		
*Socio-economic Characteristics*		
Place of Residence		
Geographical Locations	Separate dummy variables for Western Province, Central Province, Southern Province, Northern Province, Eastern Province, North Eastern Province, North Central Province, Uva Province And Sabaragamuwa Province; Uva Province is the reference category.	(+/−)
Sectors	Separate dummy variables for urban, rural and estate. Estate is the reference category.	(+/−)
Income	Per capita Household Monthly Income (SLRs.‘000)	(−)
Household Head’s Education Level	Separate dummy variables for no-schooling, primary, secondary, tertiary, higher and special. Special is the reference category.	(+/−)
Sources of Drinking Water	Separate dummy variables for protected well-within premises, protected well-outside premises, unprotected well, tap inside home, tap within premises, tap-outside premises, project in village, tube well, bowser, river/tank/streams, rain water, bottled water, other. Rainwater is the reference category.	(+/−)
Available Toilet Facilities	Separate dummy variables for water seal-connected to pit, water seal-connected to drainage system, not water seal, direct pit, and Other. Other is the reference category.	(+/−)
Household Head’s Employability Status	Separate dummy variables for government, semi government, private, own account worker, and contributing family worker. Contributing family worker is the reference category.	(+/−)
*Demographic Characteristics*		
Household Head’s Age	Age of the household heads (in years)	(+/−)
Religion	Separate dummy variables for Buddhist, Hindu, Islam, and other. Other is the reference category.	(+/−)
Ethnicity	Separate dummy variables for Sinhalese, Sri Lankan Tamil, Indian Tamil, Sri Lankan Moors, and other. Other is the reference category.	(+/−)

Source: Authors’ compilation

## Results

The estimated results are based on the logit model with the aim of achieving the objective of the study, i.e. investigate the socio-economic and demographic characteristics associated with child mortality. Therefore, the study results take into account: 21,756 household units, 147 households who has experienced child deaths as a level of 0.68% of the absolute sample populace accounted for in 2016 in Sri Lanka. Table 3 captures and summarises the basic characteristics of above-mentioned details.

**Table 3:** Characteristics of Sri Lankan households 2016

Variable	Analytical Sample (***N*** = 21,756)	
	% (Means if numerical)	Std. deviation
*Dependent Variable*		
*Child Mortality*		
Yes	0.68%	
No	99.32%	
*Independent Variables*		
*Socio-economic Characteristics*		
*Place of Residence*		
Province-Wise		
Western	22.73%	
Central	12.70%	
Southern	14.68%	
Northern	9.21%	
Eastern	8.99%	
North Eastern	10.45%	
North Central	6.27%	
Uva	6.04%	
Sabaragamuwa	8.93%	
Sector-Wise		
Urban	15.76%	
Rural	79.95%	
Estate	4.29%	
*Income*	16.2915	23.7123
*Household Head’s Education Level*		
Non-schooling	3.42%	
Primary	22.81%	
Secondary	22.68%	
Tertiary	48.12%	
Higher	2.93%	
Special	0.03%	
Sources of Drinking Water		
Protected well-within premises	32.24%	
Protected well-outside premises	11.25%	
Unprotected well	2.60%	
Tap inside home	25.83%	
Tap within premises	5.64%	
Tap-outside premises	1.69%	
Project in village	7.12%	
Tube well	3.61%	
Bowser	0.77%	
River/Tank/Streams	5.67%	
Rainwater	0.09%	
Bottled water	0.62%	
Other	2.87%	
Toilet facility type		
Water seal-connected to pit	92.42%	
Water seal-connected to drainage system	3.54%	
Not water sealed	1.45%	
Direct pit	1.46%	
Other toilet types	0.14%	
*Household Head’s Employability Status*		
Government	36.63%	
Semi-government	2.21%	
Private	30.26%	
Employer	2.11%	
Own account worker	28.33%	
Unpaid family worker	0.47%	
*Demographic Characteristics*		
Household Head’s Age	52.62	14.05
*Religion*		
Buddhist	68.64%	
Hindu	15.30%	
Islam	8.59%	
Roman Catholic/Other Christian	7.46%	
Other	0.01%	
*Ethnicity*		
Sinhalese	72.52%	
Sri Lankan Tamil	15.07%	
Indian Tamil	3.62%	
Sri Lankan Moors	8.38%	
Malay	0.22%	
Burgher	0.13%	
Other	0.05%	

Source: Authors’ compilation based on the DCS [48]

The underlying the initial logit model was assessed utilizing all independent factors and results are summarised in Table 4.

**Table 4:** Initial Logic model estimation results for household dataset, Sri Lanka

Variable	Estimate	Robust SE
Constant	−25.3767	
*Socio-economic characteristics*		
*Place of Residence*		
*Province-Wise*		
Western	−1.2157***	0.3815
Central	−0.5700	0.3571
Southern	−0.6024*	0.3615
Northern	−1.0787*	0.5520
Eastern	−0.4496	0.3943
North Eastern	−0.9396**	0.4346
North Central	−0.0616	0.4185
Sabaragamuwa	− 0.5186	0.3888
*Sector-Wise*		
Urban	−0.1022	0.6479
Rural	0.1144	0.6107
*Income*	0.0025	0.0021
*Household Head’s Education Level*		
Non-schooling	−2.7287**	1.2868
Primary	− 2.8444**	1.2341
Secondary	−2.8829**	1.2357
Tertiary	−2.7390**	1.2294
Higher	−3.0459**	1.3794
*Sources of Drinking Water*		
Protected well-within premises	−0.2494	0.4464
Protected well-outside premises	−0.4256	0.4875
Unprotected well	−0.5988	0.7139
Tap inside home	−0.1853	0.4622
Tap within premises	−1.2844**	0.6411
Tap-outside premises	−0.2670	0.7087
Project in village	−0.9767*	0.5744
Tube well	−0.6552	0.6671
Bowser	0.1272	0.8470
River/Tank/Streams	−0.3847	0.5731
Bottled water	0.5310	0.8010
*Toilet facility type*		
Water seal-connected to pit	−1.4723	1.0386
Water seal-connected to drainage system	−1.7677	1.1507
Not water sealed	−0.9379	1.1568
Direct pit	−1.3164	1.1781
*Household Head’s Employability Status*		
Government	12.4841***	0.4980
Semi-government	12.6090***	0.7448
Private	12.8297***	0.5110
Own account worker	12.6969***	0.4982
*Demographic Characteristics*		
Household Head’s Age	−0.0028	0.0077
*Religion*		
Buddhist	0.0698	0.5429
Hindu	0.1364	0.5054
Islam	0.5633	0.3515
*Ethnicity*		
Sinhalese	12.7448***	0.9925
Sri Lankan Tamil	13.2220***	0.9820
Indian Tamil	13.1307***	1.1490
Sri Lankan Moors	13.2197***	0.9126
Area under ROC curve	0.6704	
Pseudo R^2^	0.0346	
Log likelihood	−847.5612	
No. of observation	21,297	

Source: Authors’ calculation based on the DCS [48]

In the variable section for the final logit model, forward stepwise technique has used with the *p*-value < 0.10 and recently chosen factors for evacuation with *p*-value > = 0.15. For example, insignificant variables on child mortality such as sectors, government employer, Buddhist, Sinhalese were excluded when generating the final logit model for the study, which is shown in Table 5. The Receiver Operating Characteristic curve (ROC) value was 0.6494, which indicates that the final logit model fits aptly, with the aim of explaining the link between the various socio-economic and demographic characteristics on child mortality. Table 5 presents generated results of coefficients where place of residence as province-wise, household head’s education level and source of drinking water has a negative association with child mortality in Sri Lanka.

**Table 5:** Logic model estimation results for household dataset, Sri Lanka

Variable	Estimate	Robust SE	Marginal Effect
Constant	−1.6902		
*Socio-economic characteristics*			
*Place of Residence*			
Western	−1.1689***	0.2829	−0.0053
Central	−0.4221	0.2682	−0.0022
Southern	−0.5654**	0.2696	−0.0028
Northern	−0.6354*	0.3353	−0.0030
North-Eastern	−0.8516**	0.3314	−0.0037
Sabaragamuwa	−0.4654	0.3144	−0.0023
*Household Head’s Education Level*			
No Schooling	−2.8015**	1.2908	−0.0062
Primary	−2.9325**	1.2415	−0.0111
Secondary	−2.9401**	1.2437	−0.0110
Tertiary	−2.7953**	1.2346	−0.0208
Higher	−3.0703**	1.3622	−0.0062
*Sources of Drinking Water*			
Tap within premises	−1.0451**	0.5148	−0.0041
Project in village	−0.6937	0.4248	−0.0031
*Household Head’s Employability Status*			
Private	0.2662	0.1736	0.0017
*Demographic Characteristics*			
*Ethnicity*			
Sri Lankan Moors	0.6916***	0.2234	0.0056
Area under ROC curve	0.6494		
Pseudo R^2^	0.0269		
Log likelihood	− 854.2914		
No. of observation	21,297		

Source: Authors’ calculation based on the DCS [48]

## Discussion

To the extent socio-economic and demographic characteristics of households in Sri Lanka considered, most households were from the Western province with 22.73 and 79.95% from the rural sector. Most of the household heads have been educated up to tertiary level (48.12%), majority of the households have a protected wells within their premises as a source of drinking water (32.24%), most common toilet facility is water seal-connected to pit (92.42%), most of the household heads are government employees (36.63%), the average household head’s age can be taken as 53 years and finally most of the households are from Buddhism religion (68.64%) and Sinhalese ethnicity (72.52%).

The generated results of coefficients where place of residence as province-wise, household head’s education level and source of drinking water is negatively associated with child mortality in Sri Lanka. Specially, the Western province has the strongest negative association on child mortality which indicates that this province has the greatest probability of child survival compared to other provinces. Southern province, North Eastern province and Northern province also stand as leading provinces which has a negative association with child survival. Nevertheless, among these, North-Eastern province has a higher probability on child death. This finding is contradictory with the findings of S Rajindrajith, S Mettananda, D Adihetti, R Goonawardana and NN Devanarayana [10] which discovered that the Western province leads for child mortality in Sri Lanka.

The marginal effect of household head’s education level indicates that households head who studied up to tertiary level has higher safety on child survival over those household heads whose education levels are below the tertiary level. Furthermore, heads who did not attend school and those who did not study up to higher level have the lowest level of marginal effect, which denotes the lowest level of negative association on child mortality in Sri Lanka. Meanwhile, the above findings has been confirmed from the analysis conducted by [15], where household head’s level of education is significantly associated on child mortality. Furthermore, it can be highlighted that there is higher risk for child mortality in households whose head is educated up to primary level compared to those who had been educated up to secondary level and above.

According to the source of drinking water, tap within premises and project (associated with drinking water) in village have identified as significant variables on child mortality. Here, tap within premises has 0.41 probability of child survival compared to the project in village. Use of private toilets may be just as important in preventing child mortality as use of modern flush toilets and public toilets increases child mortality as well [39]. The use of bush is clearly associated with the highest risk of child mortality.

Employability status of household head as private and under the demographic category of religion Sri Lankan Moors have a positive association on child mortality as well. According to the estimated marginal effect, Sri Lankan Moors account for 0.56 percentage points higher compared to other religions in Sri Lanka. However, findings observed by [46] is contrary to the above mentioned conclusions; here, researchers noted that religion had a negative association on child mortality. Nevertheless, household heads who are employed in the private sector have 0.17 percentage points higher compared to other employability categories of household heads. This means that Sri Lankan Moors and private sector household heads have high risk on child mortality. A noteworthy review conducted in Italy suggests that unemployment affect child mortality through improvement in employment levels and economic growth will lead to reduce child mortality [19].

Province wise, the Western province recording the lowest risk can be attributed to households have access to health facilities, high level of education of household parents and as a result, they being aware of child health issues. A similar situation can be noted in Southern and Northern provinces, but latter is questionable (as socio-economics and demographics, can vary among these two provinces) and need to be followed with qualitative investigation including interviews and discussions. Referring to these findings as the base, policymakers are can make productive decision making, devise polices and special programmes to address high risk areas, allocate resources to address paediatric health issues in respective provinces.

Source of drinking water is increasingly becoming a major concern, where even at rural level, drinking water projects for households are essential. Farmers being unaware of fertiliser practices and its impacts, low quality of chemical fertiliser used, prevailing chronic kidney disease (CKD) prevailing in agriculture-based regions can be attributed to current issues arising of contaminated natural water sources. General assumption that Sri Lanka has plenty of natural water springs, wells etc., seems not valid anymore. Even rural households face shortage of water for consumption; this means, access to drinking water is an issue at the village level. Another concern is that households should be advised and be made aware on hygiene, ensure hygiene of drinking water such as boiling etc. Contaminated water tend be a breeding ground for many diseases, i.e. cause for many child health deaths.

An in depth analysis can be recommended for the North Eastern province regarding policy implications targetting Moors, where the highest child mortality rates are reported. For Sri Lankan Moors ethnicity group, whether there is a link between toilet facilities of households and source of drinking water towards child death, is worthwhile investigating. In future studies, this can be further extended combining the Eastern province.

Another concern that tend to go unnoticed is good health habits of households such as having toilet facilities in households. Some household can afford but fail to understand that having hygienic toilet facilities is not merely a basic need but also one that is essential. Hence, poor attention is given to toilet facilities and hygiene. Again, unsafe toilet practices and poor toilet facilities at homes can cause many health related disorders. This is even more critical for infants and children during age 0–5, as they are prone to catch disease during this time.

Some important variables may have not been considered in this study. For example, distance to the nearest main hospital which are in a position to care for child healthcare services is also a concerns not captured in the present study. Regardless of implementing a solution which ‘fits all’, following rules and procedures as in typical government administration in Sri Lanka, each region and region-specific issues may be handled successfully on a case by case basis. This will be an eye opener and a significant change with regard to outlook of administrators as well. Policymakers need to look beyond the ‘need for funds’ or the ‘larger slice of the budget’. Instead, this issue needs to address these health myths, misconceptions and lack of awareness on simple aspects that can make a significant change – to mark a turning point in policies and strategies in reducing child mortality.

The purpose of this study is to investigate the socio-economic and demographic characteristics associated with child mortality in Sri Lanka. On top of this, generated results emphasise that employability status of household head as in the private sector and under the demographic category of religion Sri Lankan Moors have a positive association on child mortality can be considered as risk factors on child deaths; place of residence as province-wise, household head’s education level and source of drinking water has been identified as variables which generate a negative association on child mortality in Sri Lanka, which positively associate with child survival.

## Conclusions

This study discovered that the place of residence province wise, family unit head’s education level and source of drinking water has negative effect on child mortality (i.e. lower risk on child mortality) in Sri Lanka. In other words, these factors relatively have a lower association on child mortality. A unique finding is that the Western region has the highest negative effect on child mortality which shows that it is the area with the least risk in Sri Lanka in child endurance. With regard to household head’s education level, the heads who had received education up to higher level has the lowest risk on child mortality when compared to others. When government provide subsidy programmes on child welfare and survival, the above mentioned factors can be taken into account in decision making. At this point, priority can be given to less privileged groups, i.e. such as households whose heads have received education up to higher level. Concerning the source of drinking water, comparatively to other sources, households with taps within premises have the lowest risk on child survival. Due to this reason, health sector, policymakers or any other responsible institutions need to give priority and proper attention on other households when providing knowledge on child survival.

A key benefit of this study is that it resembles a practical flavour. Government and responsible institutions will be in a better position to comprehend the real-world consequences and impacts, i.e. to identify which zones they need to give more thought on child endurance, specially in a pandemic like COVID-19. In such circumstances, country needs to address issues of high risk zones, allocate funds and resources and rearrange their wellbeing at workplaces to ensure overall prosperity of citizens. In the Sri Lankan setting, policymakers could plan to develop new clinical administration systems and practices that address core issues relating to child health, in order to combat the issue of child endurance. Hence, Central and Sabaragamuwa provinces have been recognised as higher peril domains for child mortality.

## Data Availability

The data that support the findings of this study are available from Department of Census and Statistics of Sri Lanka, but restrictions apply to the availability of these data, which were used under license for the current study, and so are not publicly available. Data are however available from the authors upon reasonable request and with permission of Department of Census and Statistics of Sri Lanka.

## Abbreviations

ABDC: Australian Business Deans Council
CHD: Chronic kidney disease
COVID-19: Coronavirus
HIES: Household Income and Expenditure Survey
UN: United Nations
UNICEF: United Nations Children’s Fund
UNIGME: United Nations Interagency Group for Child Mortality Estimation
WHO: World Health Organization

## References

[1] Hobcraft JN, McDonald JW, Rutstein SO (1984). Socio-economic factors in infant and child mortality: a cross-national comparison. Popul Stud.

[2] Veneman AM (2007). Education is key to reducing child mortality: the link between maternal health and education. UN Chron.

[3] Children: Improving Survival and well-being [https://www.who.int/news-room/fact-sheets/detail/children-reducing-mortality]. Accessed 30 Oct 2020.

[4] Ezeh O, Agho K, Dibley M, Hall J, Page A (2015). Risk factors for postneonatal, infant, child and under-5 mortality in Nigeria: a pooled cross-sectional analysis. BMJ Open.

[5] Mogford L (2004). Structural determinants of child mortality in sub-Saharan Africa: a cross-national study of economic and social influences from 1970 to 1997. Soc Biol.

[6] United Nations Inter-agency Group for Child Mortality Estimation (2018). Levels and Trends in Child Mortality Report 2018: Estimates developed by the UN Inter-agency Group for Child Mortality Estimation.

[7] Trussell J, Hammerslough C (1983). A hazards-model analysis of the covariates of infant and child mortality in Sri Lanka. Demography.

[8] Child and Infant Mortality [https://ourworldindata.org/child-mortality]. Accessed 8 Nov 2020.

[9] Houweling T, Ronsmans C, Campbell O, Kunst A (2007). Huge poor-rich inequalities in maternity care: an international comparative study of maternity and child care in developing countries. Bull World Health Organ.

[10] Rajindrajith S, Mettananda S, Adihetti D, Goonawardana R, Devanarayana NN (2009). Neonatal mortality in Sri Lanka: timing, causes and distribution. J Matern Fetal Neonat Med.

[11] Yanikkaya H, Selim S (2010). The determinants of infant mortality in Turkey: a disaggregated analysis. İktisat İşletme ve Finans.

[12] Babayara M, Addo B (2018). Risk factors for child mortality in the Kassena-Nankana District of northern Ghana: a cross-sectional study using population-based data. Scientifica.

[13] Yanıkkaya H, SelİM S (2010). The determinants of infant mortality in Turkey: a disaggregated analysis. Iktisat Isletme ve Finans.

[14] Iram U, Butt Muhammad S (2008). Socioeconomic determinants of child mortality in Pakistan: evidence from sequential probit model. Int J Soc Econ.

[15] Kaberuka W, Mugarura A, Tindyebwa J, Bishop D (2017). Factors determining child mortality in Uganda. Int J Soc Econ.

[16] Rabbani S, Qayyum A (2018). Comparative analysis of factors affecting child mortality in Pakistan. Res J Soc Sci.

[17] Kembo PJ, Ginneken J (2009). Determinants of infant and child mortality in Zimbabwe: results of multivariate hazard analysis. Demogr Res.

[18] Choe M, Luther N, Pandey A, Chand J (1999). Identifying children with high mortality risk. Nat Fam Health Survey Bull.

[19] Dallolio L, Di Gregori V, Lenzi J, Franchino G, Calugi S, Domenighetti G, Fantini MP (2012). Socio-economic factors associated with infant mortality in Italy: an ecological study. Int J Equity Health.

[20] Mondal MN, Kamal M, Korban A (2009). Factors influencing infant and child mortality: a case study of Rajshahi District, Bangladesh. J Hum Ecol.

[21] Khan JR, Awan N (2017). A comprehensive analysis on child mortality and its determinants in Bangladesh using frailty models. Arch Public Health.

[22] Pörtner C, Tarp F, Kovsted J (2002). Child health and mortality: does health knowledge matter?. J Afr Econ.

[23] Pandey A (1998). Infant and child mortality in India.

[24] Apunda R (2016). Determinants of child mortality in Kenya.

[25] Victora CG, Barros AJD, Blumenberg C, Costa JC, Vidaletti LP, Wehrmeister FC, Masquelier B, Hug L, You D (2020). Association between ethnicity and under-5 mortality: analysis of data from demographic surveys from 36 low-income and middle-income countries. Lancet Glob Health.

[26] Schulpen TWJ, van Steenbergen JE, van Driel HF (2001). Influences of ethnicity on perinatal and child mortality in the Netherlands. Arch Dis Child.

[27] Adedini SA, Odimegwu C, Imasiku ENS, Ononokpono DN (2015). Ethnic differentials in under-five mortality in Nigeria. Ethnicity Health.

[28] Caldwell J, McDonald P (1982). Influence of maternal education on infant and child mortality: levels and causes. Health Policy Educ.

[29] Gaisie SK (1975). Levels and patterns of infant and child mortality in Ghana. Demography.

[30] Genowska A, Jamiolkowski J, Szafraniec K, Stepaniak U, Szpak A, Pajak A (2015). Environmental and socio-economic determinants of infant mortality in Poland: an ecological study. Environ Health.

[31] Abu IN, Madu IA, Ajaero CK (2015). The prevalence and determinants of under-five mortality in Benue state, Nigeria. SAGE Open.

[32] Durkin MS, Davidson LL, Kuhn L, O’Connor P, Barlow B (1994). Low-income neighborhoods and the risk of severe pediatric injury: a small-area analysis in northern Manhattan. Am J Public Health.

[33] Ssewanyana S, Younger SD (2007). Infant mortality in Uganda: determinants, trends and the millennium development goals. J Afr Econ.

[34] Kimani M (2002). Behavioural effects of infant and child mortality on fertility in Kenya. Afr J Reprod Health.

[35] Nyamuranga C, Shin J (2019). Public health expenditure and child mortality in southern Africa. Int J Soc Econ.

[36] Stella Lartey RK (2016). Shingo Takahashi: the impact of household wealth on child survival in Ghana.

[37] Shiferaw Y, Zinabu M, Abera T. Determinant of infant and child mortality in Ethiopia: SSRN Electronic Journal; 2012.

[38] Saha UR, van Soest A (2013). Contraceptive use, birth spacing, and child survival in Matlab, Bangladesh. Stud Fam Plan.

[39] Stephens PW (1985). The relationship between the level of household sanitation and child mortality - an examination of ghanaian data. Afr Demography Working Paper Series.

[40] Odimegwu CO, Olamijuwon EO, Chisumpa VH, Akinyemi JO, Singini MG, Somefun OD (2020). How soon do single mothers have another child? A competing risk analysis of second premarital childbearing in sub-Saharan African countries. BMC Pregnancy Childbirth.

[41] Akinyemi J, Solanke B, Odimegwu C (2018). Maternal employment and child survival during the era of sustainable development goals: insights from proportional hazards modelling of Nigeria birth history data. Ann Global Health.

[42] Tripathi V, Singh R (2015). Ecological and socio-demographic differences in maternal care services in Nepal. PeerJ.

[43] Bello RA, Joseph AI (2014). Determinants of child mortality in Oyo state, Nigeria. Afr Res Rev.

[44] Okwaraji YB, Cousens S, Berhane Y, Mulholland K, Edmond K (2012). Effect of geographical access to health facilities on child mortality in rural Ethiopia: a community based cross sectional study. PLoS One.

[45] Poppel FV, Schellekens J, Liefbroer AC (2002). Religious differentials in infant and child mortality in Holland, 1855–1912. Popul Stud.

[46] Mutunga C, Mcgillivray M, Dutta I, Lawson D (2011). Environmental determinants of child mortality in Kenya. Health Inequality and Development.

[47] Ethnicity and child mortality in sub-Saharan Africa [https://knowledgecommons.popcouncil.org/cgi/viewcontent.cgi?article=1251&context=departments_sbsr-pgy]. Accessed 7 Aug 2020.

[48] DCS, Department of Census and Statistics SL (2016). Household income and expenditure survey 2016.

